# Decision Not to Realise Capital

**Published:** 1920-08-14

**Authors:** 


### METROPOLITAN HOSPITAL SUNDAY FUND.

### Decision Not to Realise Capital.

A meeting of the Council of the Metropolitan Hospital Sunday Fund was held on Thursday, August 5, at the Mansion House, to receive the report of the Committee of Distribution for the current year and to order payment of the awards it recommended. These documents, which were published in our last week's issue, pp. 492-493, were passed and sanctioned.

Amongst those present were the Vice-President (Mr. R. Holland-Martin), in the chair; Sir William Church (Chairman of the Distribution Committee), Lord Somerleyton, Sir George Makins, Viscount Knutsford, Sir Dyce Duckworth, the Rev. W. Hardy Harwood, Mr. John H. Buxton, and Mr. Arnold James (Secretary).

The Chairman (**Mr. R. Holland-Martin**), Vice-President of the Fund, moved the adoption of the report. He said the proceeds of their collections available for distribution amounted to £110,000, which was £25,000 higher than the previous record of £85,000 in 1918. When everything had come in the total sum received would be nearer £112,000. At the beginning of the year, when they started collecting, they were told that they would be met by many special difficulties, that heavy taxation and the higher cost of living would cause people to give less than they usually gave, and that there would be a disposition on the part of the charitable public to expect the working man to pay a great deal more towards the upkeep of hospitals. He was glad to say they had found that the working man had provided a considerably increased sum. A large sum had been collected in pence, and in many working-class districts of London, such as Hoxton, very good collections were made. He did not think there was any need to fear that the working man would not support the Fund. The Lord Mayor had given the Fund valuable help in a special way. He made a "drive" amongst business houses, and at Lloyd's, the Stock Exchange, and the Baltic. It was due to him and the energy of its Chairman that Lloyd's had contributed no less than £5,045. The Baltic and the Stock Exchange had also opened special collections, which had been generously subscribed to, and large increases were recorded. A great deal of help had also come from business houses, several of which gave large amounts. He was confident that good results would be obtained if large business houses would open collections in their offices. Another body he wished to thank was the London branch of the Red Cross, which this year collected just short of £1,500. Many of the churches had done a great deal better, and of those in the poorer districts St. Andrew's, Lambeth, had collected £147; St. Ann's, Stamford Hill, £155; St. John's, Hoxton, £9[illegible] Whitechapel Primitive Methodist, £150; St. Jude's, Mil[illegible] may Park, £128; and Hoxton Market Mission, £100. Th[illegible] practice of house-to-house collection had extended, an[illegible] promised to extend further. Though they had achieved [illegible] record this year, the Fund would not rest content with i[illegible] and he was hopeful that in another year they wou[illegible] exceed it.

**Viscount Knutsford** drew attention to a paragraph [illegible] the report which spoke of a special meeting of the Di[illegible] tribution Committee on July 6 last to consider the de[illegible] sirability of increasing grants to hospitals out of capit[illegible] He said that in view of the crisis that is likely to ar[illegible] next year, and the possibility of some of the larger ho[illegible] pitals being compelled to close, it seemed to him a pi[illegible] if, possessing the capital, they did not in an emergen[illegible] go to the assistance of an institution, and by a speci[illegible] grant save it from the danger of having to close dow[illegible] He expressed a desire to move a resolution which wou[illegible] empower the Distribution Committee to call a special mee[illegible] ing to consider the making of special grants out of capi[illegible] if, in a crisis, a hospital is faced with a position whi[illegible] must result in closing.

In answer to questions, the Secretary (Mr. Arno[illegible] James) said that the Fund has a capital of £800,000, b[illegible] probably a realisation would produce only betwe[illegible] £500,000 and £600,000.

**Sir William Church**, replying to Viscount Knutsfor[illegible] said that the matter raised by Lord Knutsford had be[illegible] considered, and at the special meeting of the Committ[illegible] on July 6 it was decided not to recommend grants fr[illegible] capital this year. It was felt that the Fund was f[illegible] maintenance, and to make a grant to any one single ho[illegible] pital that happened to be most in debt would hit ot[illegible] hospitals. By sacrificing a portion of their capital th[illegible] would be diminishing the grants, and on the whole t[illegible] hospitals would suffer.

**Viscount Knutsford** said the whole question was a ve[illegible] difficult one, because they did not want to save one ho[illegible] pital at the expense of the others. This year was t[illegible] most critical year that hospitals would ever have to [illegible] If prices remained at their present level the London H[illegible] pital could not possibly go on. He withdrew his prop[illegible] and would not move a resolution, contenting himself w[illegible] having drawn attention to the matter.

The report was then adopted, **Sir Dyce Duckwo[illegible]** thanking the Distribution Committee, on behalf of t[illegible] Council, for their care in the preparation of the award[illegible]

